# Supplementary material for: Auxin-Induced SaARF4 Downregulates SaACO4 to Inhibit Lateral Root Formation in Sedum alfredii Hance
Source: Int J Mol Sci. 2021 Jan 28;22(3):1297. doi: 10.3390/ijms22031297 (PMC7865351; doi:10.3390/ijms22031297)
Supplement: Supplementary file 1 [file ijms-22-01297-s001.zip › supplymental file.docx]

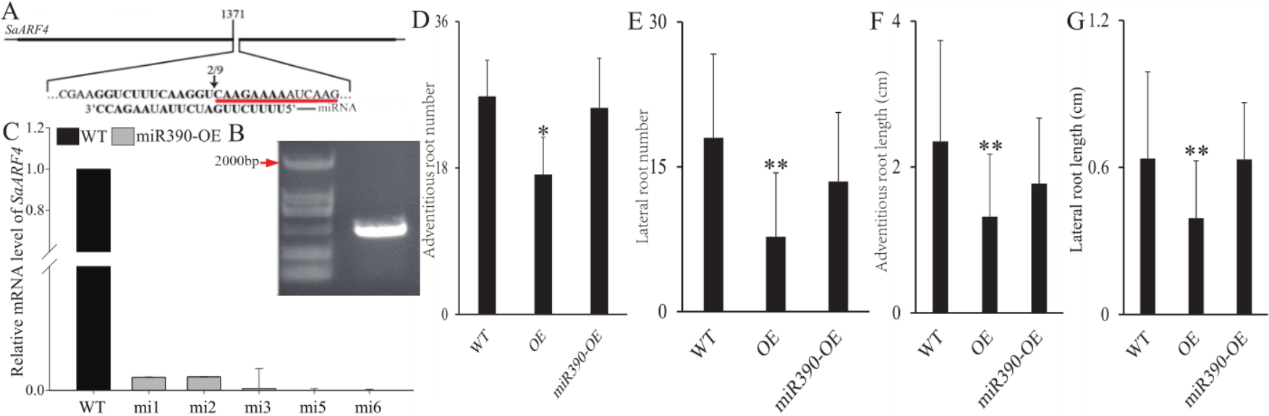


**Figure S1 Overexpression of *miR390* did not change LRs development of HE**

**A** The cleavage site of *miR390* in *SaARF4* (verified by the technology of RLM-RACE); **B** The clone of MIR390; **C** Overexpression of *miR390* decreased the expression levels of *SaARF4* in different transgenic lines; **D-G** the comparison of root number and length among WT, OE and miR390-OE; *, P <0.05; **, P <0.01


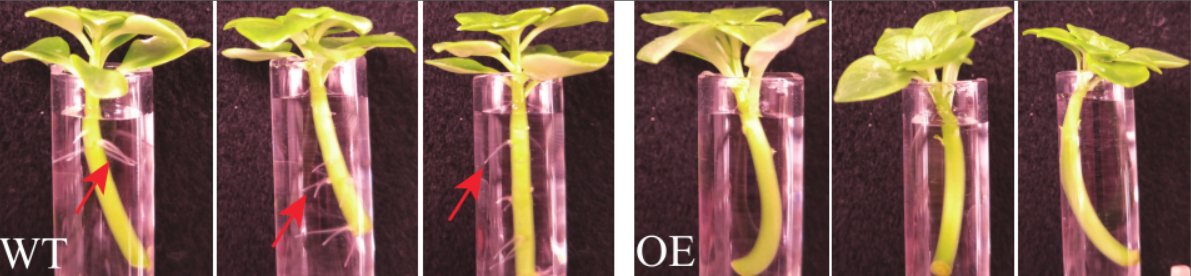


**Figure S2 The comparison of rooting rates between WT and OE**

The different lines of **WT** and **OE** with similar growth situation were cultured about seven days in hydroponics. We found that the generation of LRs (the red arrows) of WT was faster than those of OE.


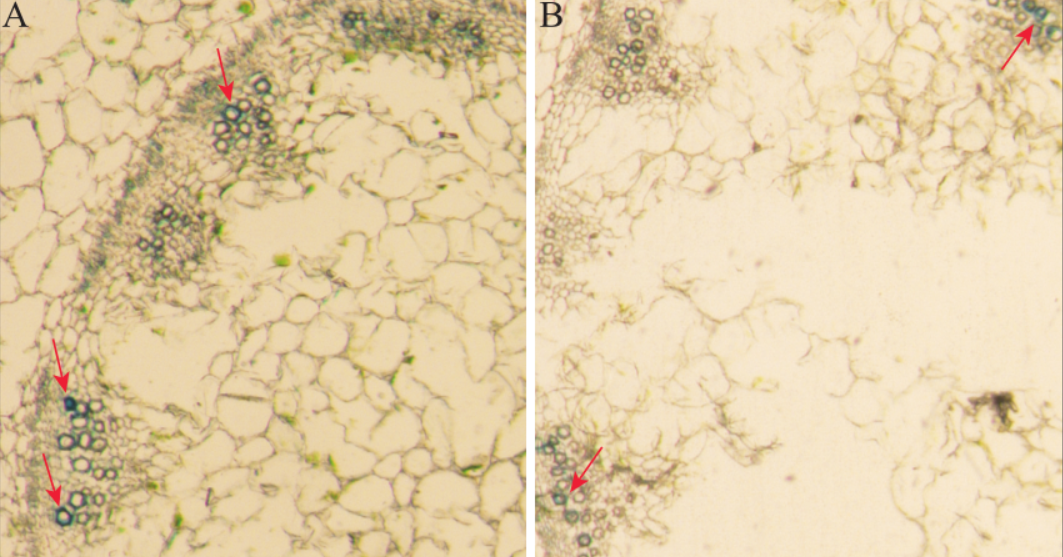


**Figure S3 The distribution of GUS signals in xylem of *ProARF4::GUS* different transgenic lines**

**A** and **B** indicated different transgenic lines of *ProARF4::GUS*; The red arrows indicated the areas that *ProARF4::GUS* expressed in.


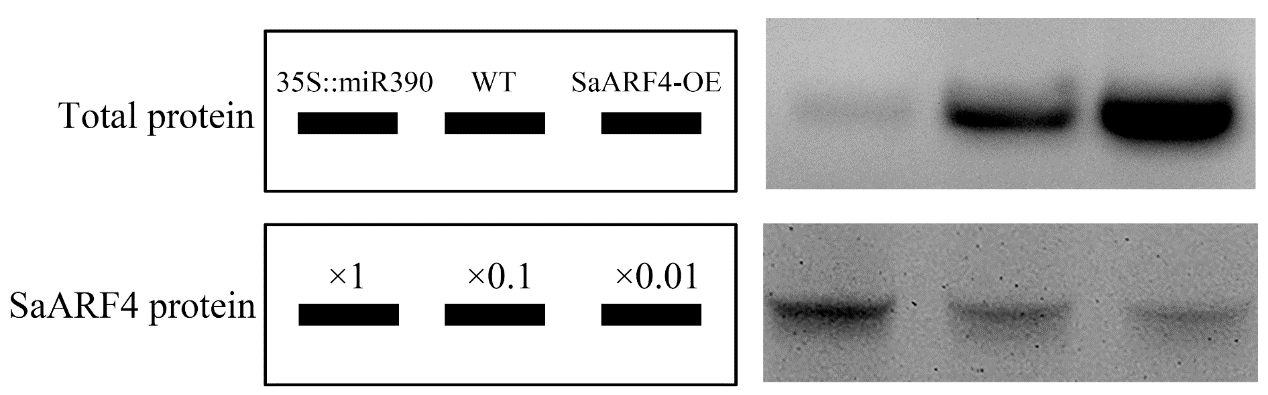


**Figure S4 Western blot**

The western blot was conducted as Figure S4 showed, with 100mg total protein and 30 μg SaARF4 protein. Total protein was extracted from WT, SaARF4-OE and *35S::miR390* plants, respectively. SaARF4-protein amounts were diluted as indicated in the figures.

**Table S1 Primers**

| Primers | Sequences (5′-3′) | Descriptions |
| --- | --- | --- |
| UBC9-F | TGGCGTCGAAAAGGATTCTGA | qRT-PCR |
| UBC9-R | CCTTCGGTGGCTTGAATGGATA | qRT-PCR |
| SaARF4-F | GCTGGAATGGCTGTTTGGAA | qRT-PCR |
| SaARF4-R | CTCCACCTCTTCTTCCGTGT | qRT-PCR |
| SaACO2-F | CGTGCTACACCGGGTTATTG | qRT-PCR |
| SaACO2-R | TTGACGCGCAGATAAAGCTC | qRT-PCR |
| SaACO4-F | TGTCCCAAGCCACATCTGAT | qRT-PCR |
| SaACO4-R | CTGTTGGCGCATTTACTCGA | qRT-PCR |
| SaACO4.2-F | CCTGCGAAAACTGGGGATTC | qRT-PCR |
| SaACO4.2-R | CCATGTCAGTGACCTCAGCT | qRT-PCR |
| SaACO5-F | GAGGACACGAGCGTTTCTTT | qRT-PCR |
| SaACO5-R | TGAGTATTTGTAGGCCGCCA | qRT-PCR |
| SaACO5.2-F | CAGGCGGCGTCATACTACTA | qRT-PCR |
| SaACO5.2-R | CTAAGGCTAAAACCCGGTGC | qRT-PCR |
| SaPIN1-F | ACAACAACGTCGGTCCAAAG | qRT-PCR |
| SaPIN1-R | ATTATCTTCGACGCTCCGGT | qRT-PCR |
| SaPIN1.2-F | ACCGTTTCGTTGCACTCTTC | qRT-PCR |
| SaPIN1.2-R | ACCATTCAAGCGAGCCTCTA | qRT-PCR |
| SaPIN1.3-F | TGCAATTCCCTCGGTTACCT | qRT-PCR |
| SaPIN1.3-R | GCCAAGATTAACACCCGCTT | qRT-PCR |
| SaPIN2-F | AATCTTCACCCCGGACCAAT | qRT-PCR |
| SaPIN2-R | GCCAAAGAAACAGAGCGGAA | qRT-PCR |
| SaPIN2.2-F | ATCCCTTTGGTCCGGAATGT | qRT-PCR |
| SaPIN2.2-R | TGCTGCTCCTCAACCTGAAT | qRT-PCR |
| SaPIN2.3-F | AGAGATCATGGGTGGTGCAA | qRT-PCR |
| SaPIN2.3-R | CTTGGCATCGTGTACTGCAT | qRT-PCR |
| SaPIN3-F | TGATGGGTGTACAGGGCTTT | qRT-PCR |
| SaPIN3-R | CAGCAGATGACGCGATTTCA | qRT-PCR |
| SaPIN3.2-F | TGGCCATGTTTAGTCTCGGT | qRT-PCR |
| SaPIN3.2-R | AAGTGCAGCCTGAACAATGG | qRT-PCR |
| SaPIN4-F | GAGCGACGTATGCCATGATC | qRT-PCR |
| SaPIN4-R | GATGGACGCCGTACTCCTTA | qRT-PCR |
| SaPIN5-F | TGGAGCTTGATGAAGACGGT | qRT-PCR |
| SaPIN5-R | GTTGTGCCATGAACATTCCC | qRT-PCR |
| SaPIN5.2-F | GGCTGTTGGTGGTTTTGCTA | qRT-PCR |
| SaPIN5.2-R | CCAAAGATAACCGCAGTGCT | qRT-PCR |
| SaPIN6-F | TGGGTTGGAGCAATCATCCT | qRT-PCR |
| SaPIN6-R | AGAGCTTTCATCCTTCCCGT | qRT-PCR |
| SaPIN6.2-F | TGGAATCGGGAACATTGGGA | qRT-PCR |
| SaPIN6.2-R | AGGAGGTGCAAGCATACTGT | qRT-PCR |
| SaPIN6.3-F | TCAATGTGGTTCTGGGCTCT | qRT-PCR |
| SaPIN6.3-R | AACGGGTTGGATTGGTCTCT | qRT-PCR |
| SaPIN7-F | TGGAGGCATTCTTGGATGGT | qRT-PCR |
| SaPIN7-R | ATGTAGAAACCACCAAGCGC | qRT-PCR |
| SaPIN7.2-F | TCTTTGTTTGAGGTGGCTGC | qRT-PCR |
| SaPIN7.2-R | GCAAGCGATGGAGTGAACAA | qRT-PCR |
| GUS-F | CATGTTCATCTGCCCAGTCG | qRT-PCR |
| GUS-R | TGTGGGCATTCAGTCTGGAT | qRT-PCR |
| ProSaARF4-F | AGTTGTTGGCGGAAGAGAGA | GUS |
| ProARF4-R | CATGAAACCCTGCTTCCAAT | GUS |
| SaARF4-F | ATGGAAATTGAAGGGATTCATG | gene clone |
| SaARF4-R | TTATATGCAGAACTCGGTGAG | gene clone |
| pMAL-SaARF4-F | TCACATATGTCCATG ATGGAAATTGAAGGGATTCATG | Protein expression |
| pMAL-SaARF4-R | ATTTAATTACCTGCA TTATATGCAGAACTCGGTGAG | Protein expression |
| SaARF4-Sk-F | TGGATCCCCCGGGCTGCAGGATGGAAATTGAAGGGATTCATG | dual-LUC effector system |
| SaARF4-Sk-R | CGAGGTCGACGGTATCGATATTATATGCAGAACTCGGTGAG | dual-LUC effector system |
| ProSaACO4-F | GGTGTTGTTGCTTGCTTGTG | Promoter clone |
| ProSaACO4-R | ATTGCCTCAACTTGCCACTG | Promoter clone |
| ProSaACO4-LUC-F | TCGACGGTATCGATA GGTGTTGTTGCTTGCTTGTG | dual-LUC reporter system |
| ProSaACO4- LUC-R | GCTCTAGAACTAGTG ATTGCCTCAACTTGCCACTG | dual-LUC reporter system |
| ProSaACO4m1-F | GGTGTTGTTGCTTGCTTGTG | Overlapping PCR |
| ProSaACO4m1-R | GCTAATATGATTATGTAGTT | Overlapping PCR |
| ProSaACO4m2-F | AACTACATAATCATATTAGCATAACAACGAAAATAATTCA | Overlapping PCR |
| ProSaACO4m2-R | ATTGCCTCAACTTGCCACTG | Overlapping PCR |
| ProSaACO4m-LUC-F | TCGACGGTATCGATA GGTGTTGTTGCTTGCTTGTG | dual-LUC reporter system |
| ProSaACO4m- LUC-R | GCTCTAGAACTAGTG ATTGCCTCAACTTGCCACTG | dual-LUC reporter system |
| SaARF4-AD-F | CAGAGTGGCCATTATGGCCCATGGAAATTGAAGGGATTCATG | Y1H |
| SaARF4-AD-R | TCTGCAGCTCGAGCTCGATGTTATATGCAGAACTCGGTGAG | Y1H |
| SaACO4-HIS-F | CCGGTTGTTATTGTCTCGCTAATATTGTTATTGTCTCGCTAATATTGTTATTGTCTCGCTAATA TTGTTATTGTCTCGCTAATAGC | Y1H |
| SaACO4-HIS-R | TATTAGCGAGACAATAACAATATTAGCGAGACAATAACAATATTAGCGAGACAATAACAATATTAGCGAGACAATAACAA | Y1H |
